# Digital devices for teaching cardiac auscultation - a randomized pilot study

**DOI:** 10.1080/10872981.2018.1524688

**Published:** 2018-11-30

**Authors:** Malcolm E. Legget, MeiYen Toh, Andries Meintjes, Sarah Fitzsimons, Greg Gamble, Robert N. Doughty

**Affiliations:** a Department of Medicine, University of Auckland, Auckland, New Zealand; b Green Lane Cardiovascular Service, Auckland City Hospital, Auckland, New Zealand; c Institute of Biomedical Technologies, Auckland University of Technology, Auckland, New Zealand

**Keywords:** Digital stethoscope, hand-held echocardiography, cardiology, auscultation training

## Abstract

**Background**: Competent cardiac auscultation is a declining skill. Digital stethoscopes and hand-held echocardiography (HHE) are modern devices which may improve the accuracy of heart murmur recognition and diagnosis. Their incremental value compared to conventional examination has not been evaluated in depth.

**Objectives**: Our aim was to quantify the utility of digital stethoscopes and HHE as teaching aids to improve medical students’ diagnostic accuracy in the evaluation of heart murmurs using a novel clinically weighted scoring system.

**Design**: This pilot study involved eight medical students and eight patients with heart murmurs. Four patients were examined at 2 sessions, 1 week apart. Medical students were randomised into two groups: the ‘intervention group’ examined patients with a standard and digital stethoscope, and then received demonstration of the valvular lesion with HHE to illustrate the diagnosis. The ‘control group’ used a standard stethoscope only and were taught using traditional methods. Students’ scores were compared to a ‘gold standard’ derived from a consensus of auscultation findings of three cardiologists.

**Results**: Overall the mean percent correct of total possible score was 65.4% (SD8.4). Using a mixed models ANOVA approach to repeated measures, the mean [95% CI] increase from training to validation period for the control group was 2.5% [−11.5, 16.5] *P*_(Tukey)_ = 0.95 and 15.8% [1.7,29.8] *P*_(Tukey)_ = 0.027 for the intervention group. Between the validation and training sessions for both groups, there was an increase of 9.1% [1.82, 16.4] in scores (*p* = 0.018). The mean [95% CI] difference in scores of the control and intervention groups was 1.9% [−5.4, 9.2] (*p* = 0.59). The Cohen’s effect size estimate was 0.9.

**Conclusion**: Digital stethoscopes and hand-held echo may be useful devices for teaching cardiac auscultation. This pilot study provides a novel study design, a heart murmur grading system, and data that will help develop definitive studies to assess new teaching techniques for cardiac auscultation using digital technology.

## Introduction

Digital stethoscopes and hand-held echocardiography (HHE) devices are modern portable digital devices that allow physicians to evaluate patients with heart murmurs. Digital stethoscopes provide improved sound quality and the ability to record and play back sounds multiple times to multiple listeners [1]. HHE devices provide real-time visual display of cardiac valvular pathology at the patient bed-side. While well established as tools that help in the clinical assessment of patients, these tools also have the potential to facilitate effective teaching of cardiac auscultation, a core skill integral to clinical medicine. A conventional stethoscope’s inability to act as an ‘audio platform’ may be a significant obstacle to the effective teaching of cardiac auscultation [1]. The use of digital stethoscopes and HHE as teaching aids to improve the diagnostic accuracy of evaluation of heart murmurs, compared to conventional bedside examination, has not yet been evaluated in depth.

Current skills in cardiac auscultation are suboptimal [2,3]. This deficiency begins in medical school and continues after graduation [4,5]. Multiple studies have found that clinical experience does not correlate with competency in cardiac auscultation [6–9]. Fortunately, cardiac auscultation is a skill that can be taught. Teaching interventions involving repetition of heart sounds, such as with recordings and multimedia tools, have been found to effectively improve auscultatory skills [10–13], but teaching that is solely theoretical is thought to be ineffective [14].

The digital stethoscope allows immediate and pre-recorded stored playback of sounds to multiple listeners, even at reduced speeds without distortion. It features external noise filtering and the ability to amplify sound. There is also the availability of visual display allowing the localisation of sounds heard in the cardiac cycle [1,15,16]. This can allow the development of heart sound databases for continued reference and review. The portability of digital stethoscopes facilitates ease of teaching in multiple settings to larger groups of students and the accuracy of clinical examination may be increased [16].

HHE devices are miniature compact battery powered echocardiographic systems and may also be useful as a teaching tool. Advantages include their simplicity of use, ease of transport and availability at the bedside, as well as their relatively low cost [17]. HHE are potentially useful in a wide range of clinical conditions and settings. They have been found to be associated with improved detection of cardiac pathology [18], unknown cardiac disorders [19], and assessment of cardiac anatomy and function [20–22] including left ventricular hypertrophy [23], and abdominal aortic aneurysm [24,25]. There is also a growing area of literature to support that learning to use HHE can be taught quickly and effectively, even to non-cardiology medical personnel such as medical residents and students [26–31]. These positive findings suggest that HHE may be of benefit as a teaching tool for medical students and doctors in improving clinical cardiac auscultatory skills.

There is little data on the effect of teaching interventions on cardiac auscultation skills using new technology such as digital stethoscopes and HHE. A Brazilian randomised trial of 38 medical students enrolled in an 8-week cardiovascular semiology course evaluated the usefulness of digital stethoscopes (Littmann® Model 3200, 3M) compared to conventional stethoscopes in teaching auscultatory skills. The study found a much more significant improvement in the auscultation assessment score in those students who used digital stethoscopes (51.9% improvement), compared to the conventional stethoscope group (29.5% improvement) [16]. Conversely, an earlier study of 48 medical students evaluating the benefit of using a 1999 sensor-based electronic stethoscope, ‘The Stethoscope®’ over an acoustic stethoscope for a 4-month training period found no difference between the two groups of medical students with regards to their cardiac auscultation skills [32]. Another study [33] assessed a web-based training tool as well as bedside recording of heart sounds using a personal digital assistant to assist students in learning cardiac auscultation, comparing 16 students, who had trained using multimedia tools, with 13 medical residents who had received a traditional lecture in which heart sounds were played to them. The students correctly identified 80% of the heart sounds compared to 60% for the medical residents (*P* < 0.005), suggesting that the multimedia tools were effective in improving the auscultation skills of the students.

A study of training in cardiac auscultation demonstrated that repeated listening of around 500 times significantly improved the proficiency of recognition of cardiac murmurs by medical students [10]. This has led to the development of a comprehensive teaching aid called Heart Songs® promoted by the American College of Cardiology.

The aim of this pilot study was to develop a protocol to test the hypothesis that the combination of a digital stethoscope and hand-held echocardiogram-assisted teaching intervention would lead to an improvement in the cardiac auscultation skills of medical students, as compared to the use of a standard acoustic stethoscope and traditional bedside auscultation training. Further, the study aimed to validate a novel heart murmur grading system developed for measuring the accuracy of cardiac auscultation.

## Methods

This pilot study involved eight medical students. Students were recruited from volunteers that had recently finished their penultimate or started their final year of the 6-year medical degree at the University of Auckland. Cardiac auscultation is introduced in the fourth year and taught as part of the 3-year clinical curriculum, thus all participant students had received the same amount of formal auscultation training before this study. The medical students were randomly divided into a control group (four students) and an intervention group (four students). The number of students was chosen pragmatically to allow for an appropriate amount of time to be able to examine four patients in one session.

There were 2 sessions (training and validation), 1 week apart, with 4 patients examined per session (Table 1). The patients were recruited from the Green Lane Cardiovascular Service, Auckland City Hospital outpatient clinic. They were selected on the criteria of being ambulant and presenting with a cardiac murmur that had been confirmed using an echocardiogram. The patients between the two sessions were matched with similar murmurs. The patients in the training session had heart murmurs consisting of moderate mitral regurgitation, moderate pulmonary stenosis, moderate aortic stenosis, and mild aortic stenosis. The murmurs in the validation session were due to moderate mitral regurgitation, severe aortic regurgitation, and two patients with moderate aortic stenosis.

**Table 1.** Procedures followed by students in each session and arm.

Control arm	Intervention arm
**a) Training session**	
1. Examine patient with standard stethoscope for 5 min 2. Complete grading sheet 3. Lesion verbally identified	1. Examine patient with standard stethoscope for 3 min 2. Complete grading sheet 3. Examine patient with digital stethoscope for 2 min 4. Revise grading sheet 5. Lesion demonstrated using HHE
**b) Validation session**	
1. Examine patient with standard stethoscope for 5 min 2. Complete grading shee	1. Examine patient with standard stethoscope for 3 min 2. Complete grading sheet 3. Examine patient with digital stethoscope for 2 min 4. Revise grading sheet

**Figure 1.**
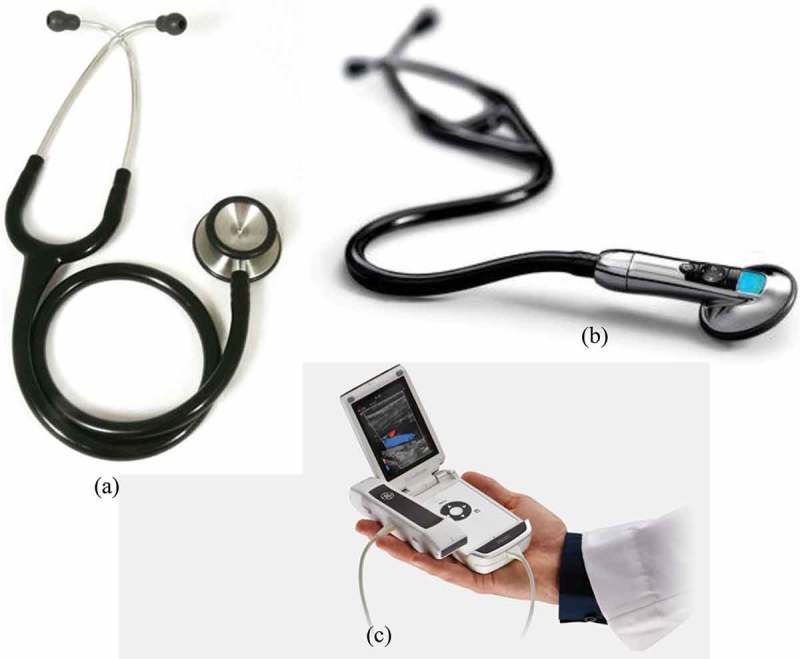
The devices used in this study. (a) A standard mechanical/acoustic stethoscope. (b) A digital/electronic stethoscope. (c) A hand-held echocardiogram device. Images reproduced under Creative Commons License. Property of Medisave.co.uk, 3M Littmann, and GE Healthcare.

The students all used a standard acoustic Littmann® stethoscope to examine the patients. The intervention group used a Littmann 3200® digital stethoscope and following grading of the murmur were then shown the echocardiogram of the patient using a General Electric Vscan® Hand-Held Echocardiography Device (Figure 1).

Data were collected using a scoring system devised by the authors incorporating auscultation findings such as murmur phase, intensity, maximal location, radiation, and change with provocative manoeuvres. Students were marked against a ‘gold standard’ derived from a consensus of auscultation findings of three cardiologists, with a weighting for each feature as indicated in Table 2. The amount of weight given to each feature was derived from the perceived importance of that feature in the overall auscultation process, for example, discerning the phase of the cardiac cycle was assigned a weighting of 5 compared with provocative manoeuvres which was assigned a weighting of 1.

**Table 2.** Heart sound scoring sheet.

Feature (weighting)				
Phase of cardiac cycle (5)	Systolic	Diastolic	Continuous	
Intensity (5)	I	II	III	IV
	V	VI		
Duration of murmur (5)	Ejection systolic	Mid systolic	Late systolic	Pansystolic
	Early diastolic	Mid diastolic		
Location heard most intense (4)	Mitral	Tricuspid	Pulmonary	Aortic
Radiation (3)	Axilla	LLSE	ULSE	URSE
	Carotid	Back	Pulmonary	Epigastrium
Character (2)	High pitched	Low pitched	Rumbling	
Added sounds (1)	None	S3	S4	Ejection click
	Systolic click			
S1 (1)	Normal	Soft	Loud	
S2 (1)	Normal	Split	Fixed Split	Reversed split
	Single			
Change with breathing (1)	Softer inspiration	Louder inspiration	Softer expiration	Louder expiration
	No change	Not done		
Valsalva (1)	Softer inspiration	Louder inspiration	Not done	
Left lateral position (1)	Louder	Softer	No change	Not done
Sitting forward (1)	Louder	Softer	No change	Not done
Final diagnosis (6)	Valve:	Severity:	Lesion:	

## Study design

### Training session (Table 1(a))

During the training session the ‘control group’ each used a standard stethoscope to examine the patients for 5 min. They then completed the rating sheet and were informed verbally of the correct diagnosis. The procedure closely resembles the traditional ward round teaching of cardiac auscultation at the University of Auckland Medical School.

The ‘intervention group’ examined patients with an acoustic stethoscope for 3 min followed by a digital stethoscope for 2 min. They recorded their diagnosis and findings after each auscultation. They were then informed of the correct diagnosis and received a demonstration of the valvular pathology using HHE by a cardiologist to illustrate the aetiology of the murmurs.

### Validation session (Table 1(b))

The same students repeated the auscultation and grading process 1 week later with a different set of 4 patients. The procedure followed was the same as during the training session for both groups.

Session one thus acted as the ‘training session’ and session two as the ‘validation session,’ with the goal being to allow comparison of scores between the two sessions and between the two groups of students.

## Statistical analysis

Since hearing and auscultation is by nature a subjective process we had to account for inter-expert variability. At each session, the ‘correct’ answer (gold standard) for each of the features on the grading sheet was chosen based on the majority finding of the three consultant cardiologists when auscultating the patients. The consultant cardiologists were blinded to the diagnoses and had to examine the patients in the same way as the students. The cardiologists’ findings were then themselves marked using the feature weighting as shown in Table 2 and the scores were averaged to calculate the total achievable score. Thus, the gold standard (and total achievable score) represents a consensus of three expert auscultators’ independent findings and the students were marked on their ability to arrive at the same, or similar, feature description. In each session, the students’ scores were averaged over the four patients examined and converted to percentages of the gold standard score.

The results were analysed to examine the changes in the accuracy of murmur grading between the control and intervention groups at each session. To explore the effect of the teaching intervention the mean of the students’ scores in each group for each session was calculated, using the results of the grading sheet for each session (step 2 for control group, step 4 for intervention group in Table 1).

Data were analysed using a mixed models ANOVA approach to repeated measures (GLM SPSS v 25, IBM). The main effects of group allocation and the repeated effect of training and validation session and their interaction were modelled. Interaction effects were further explored using Tukey’s least significance difference method to preserve an overall 5% significance level irrespective of whether the *p* value for the interaction effect was <0.05 [34]. All tests were two-tailed and *p* < 0.05 was considered significant. Cohen’s D statistic was calculated using the online calculator for ‘Effect size estimates in repeated measures designs’ provided by Psychometrica [35]. All reported confidence intervals (CI) are 95% after adjustment for multiple comparisons (Tukey method).

The study was approved by the University of Auckland and Auckland District Health Board ethics committees, approval number UAHPEC 013321, and all patients and students involved gave informed consent.

## Results

**Figure 2.**
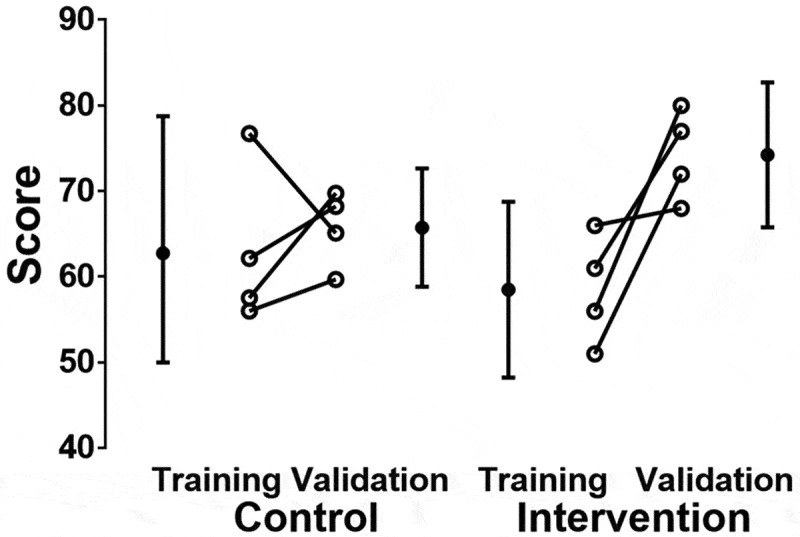
A comparison of the mean percentage score achieved between sessions for the control group and the intervention group using both standard and digital stethoscopes.

Individual comparisons of the mean and standard deviations of the control and intervention group for the training and validation sessions are shown in Table 3 and Figure 2.

**Table 3.** Comparison of mean control group scores with intervention group scores (standard and digital stethoscope). *P*-values were calculated between control and the corresponding intervention group.

Estimated marginal means for group and session^a^						
					95% Confidence interval	
Group	Session	Mean	Std. error	df	Lower bound	Upper bound
Control	Training	63.11	3.48	8	55.07	71.14
	Validation	65.70	2.12	8	60.82	70.58
Intervention	Training	58.70	3.48	8	50.66	66.73
	Validation	74.30	2.12	8	69.42	79.17

^a^Dependent Variable: Score.

Between the validation and training sessions for both groups, there was an increase of 9.1% [1.8, 16.4] in scores (*p* = 0.018). The mean [95% CI] difference in scores of the control and intervention groups was 1.9 % [−5.4, 9.2] (*p* = 0.59).

In the control arm, there was no difference between training and validation score (mean difference 2.5% (adjusted 95% CI −11.5, 16.5, *P* (Tukey post hoc protected) = 0.95). However, in the intervention arm, mean scores increased 15.8 (adjusted 95% CI 1.7, 29.8) *P* (Tukey post hoc protected) = 0.027 between the training and validation periods.

Effect size was calculated using the mean difference between training and validation scores for the control and intervention groups and the corresponding standard deviation of these differences was 0.9.

## Discussion

The ability to objectively quantify the effect of a teaching intervention, in this case to correctly identify a cardiac murmur, is complex due to the subjective nature of cardiac auscultation and the variable appreciation of the auditory qualities of a heart murmur [36].

The methodology described in this paper demonstrates a novel approach to an objective method of assessment of competency in cardiac auscultation. Although the sample size was small, the results may help to inform future study design and sample size calculation for intervention studies using digital tools as adjuncts to traditional teaching methods. There was evidence of a significant interaction between the digital devices intervention and the teaching sessions with a significant improvement in cardiac auscultation score between the training and validation phases only during the intervention period. The fact that there was no difference between overall group scores using the mixed model analysis indicates that the control and intervention groups were well matched at baseline, and any training effect was balanced. Overall, irrespective of training or intervention, the mean score was between 50% and 80% of the potential expert score suggesting room for improvement and thus the need for novel teaching approaches and study designs where these approaches can be quantitatively assessed. The effect size estimates in repeated measures designs showed that the intervention has a surprisingly large effect (*d* > 0.8), suggesting that the use of digital technology was effective in improving cardiac auscultatory skills.

The novel aspects of this pilot study relate to the combination of two digital technologies, one providing auditory and one visual feedback, as an aid to learning cardiac auscultation. Also, the development of a comprehensive cardiac auscultation grading system based on a scoring system which assigns weightings to various features of the auscultation process, for comparison between groups of auscultators, has not been reported previously to our knowledge. This enabled quantification of the various elements of cardiac auscultation so that students could be objectively assessed compared to a gold standard. This derived ‘gold standard’ may be of use when developing a teaching tool to assess the efficacy of a teaching intervention, such as the incremental application of digital auditory recordings combined with visual feedback demonstrating murmur aetiology using video recordings from HHE in this study.

The use of the stethoscope is debated in modern clinical practice, with an increase in the use of portable echocardiography devices being promoted in certain medical programs [37]. However, there are many situations when skilful auscultation can add valuable information regarding cardiac pathophysiology that is not obtainable by ultrasound, reinforcing the value of the stethoscope [38]. The design and performance of digital stethoscopes has steadily been increasing, providing better sound playback and improved user interfaces. This raises ideas about possible future study designs with larger number of students and patients, using a platform of pre-recorded heart sounds (from digital stethoscopes) and their associated HHE images as a more efficient teaching intervention.

The use of HHE has been well documented for diagnosis of a number of cardiac conditions, but not as an adjunct for cardiac auscultation training. This tool provides visual feedback at the patient bedside and immediately after auscultation. This can strengthen the student’s cognitive association between the valve lesion and the resulting heart murmur. The ability to mentally imprint the murmur with both auditory and visual feedback is likely to result in improved recognition of the sound subsequently.

The results suggest that auscultation training is positively affected by the inclusion of digital stethoscopes and HHE. The effects of such interventions require more in-depth study. In particular studies of teaching interventions that include digital devices over longer periods of time and with larger amounts of patients and students are needed for more conclusive results that can be used to inform cardiac auscultation training and teaching.

## Limitations of the study

This was a pilot study and as such the number of participants involved was small. Participant numbers were determined pragmatically to allow for an appropriate amount of time to be able to examine four patients in one session.

Some aspects of the study design may have limited the assessment of the teaching intervention. The time students were given to use the digital stethoscope was short. Their lack of familiarity with the device may have limited the advantages of its use, namely the ability to listen to the murmur multiple times. In the validation session, a practice effect may have improved murmur recognition with the use of the digital stethoscope, once students were more familiar with its use. The effect of this may have been accentuated by the design of the study since students were given the opportunity to revise their findings after using the digital stethoscope for a short time.

Students in the intervention arm may also have been disadvantaged compared to the control arm due to time constraints as they had less time to examine the patient with a standard stethoscope than control arm students (3 min compared to 5 min).

The data collection sheet has as yet not been validated. However, it was designed by an experienced cardiologist and matches schemas that have been used to describe heart sounds and murmurs found in the literature.

In terms of a practice effect, the effects of the improvement due to the use of a digital stethoscope and hand-held echo cannot be differentiated using the current study design with the small numbers of students and participants involved. This study has therefore focused on the results obtained using the digital stethoscope in the intervention arm (the revised grading sheet), rather than the standard stethoscope, to emphasise the overall effect of the combination of the use of digital devices (i.e. digital stethoscope and hand-held echo) rather than the individual effect of each technology on improving auscultation skill. In the future, introduction of the two technologies in a stepwise fashion could be incorporated into the study design to isolate the incremental teaching effect of each teaching tool.

## Conclusion

The design of studies to assess a teaching intervention is complex. Digital stethoscopes and hand-held echo demonstration of heart murmur aetiology may be useful adjuncts for teaching cardiac auscultation. This pilot study provides a novel study design, a heart murmur grading system, and data that will be useful in developing definitive studies to assess new teaching techniques for cardiac auscultation using digital technology.
